# Osteoprotegerin rich tumor microenvironment: implications in breast cancer

**DOI:** 10.18632/oncotarget.8658

**Published:** 2016-04-08

**Authors:** Sudeshna Goswami, Neelam Sharma-Walia

**Affiliations:** ^1^ H. M. Bligh Cancer Research Laboratories, Department of Microbiology and Immunology, Chicago Medical School, Rosalind Franklin University of Medicine and Science, North Chicago, Illinois, USA

**Keywords:** osteoprotegerin, NF-kB, COX-2, PGE2, FASN

## Abstract

Osteoprotegerin (OPG) is a soluble decoy receptor for tumor necrosis factor (TNF)-related apoptosis inducing ligand (TRAIL). It belongs to the tumor necrosis factor receptor superfamily (TNFRSF). OPG was initially discovered to contribute to homeostasis of bone turnover due to its capability of binding to receptor activator of nuclear factor-kappaB (NF-kB). However, apart from bone turnover, OPG plays important and diverse role(s) in many biological functions. Besides having anti-osteoclastic activity, OPG is thought to exert a protective anti-apoptotic action in OPG-expressing tumors by overcoming the physiologic mechanism of tumor surveillance exerted by TRAIL. Along with inhibiting TRAIL induced apoptosis, it can induce proliferation by binding to various cell surface receptors and thus turning on the canonical cell survival and proliferative pathways. OPG also induces angiogenesis, one of the hallmarks of cancer, thus facilitating tumor growth. Recently, the understanding of OPG and its different roles has been augmented substantially. This review is aimed at providing a very informative overview as to how OPG affects cancer progression especially breast cancer.

## INTRODUCTION

## BREAST CANCER

At present, one in eight women in the United States will develop breast cancer [1]. Recent advances in breast cancer detection and treatment have decreased the mortality rate of breast cancer [1] but the success of treatment relies largely on detection of the disease at early stages [1]. A lack of knowledge regarding the molecular mechanisms underlying breast tumor progression to invasive and then metastatic disease limits the ability to treat advanced disease. The identification of factors that promote metastasis is essential for the development of new breast cancer therapies and a further reduction in breast cancer mortality [2]. Inflammatory breast cancer (IBC) is a highly aggressive, angioinvasive, and a highly metastatic form of breast cancer. IBC is associated with a high incidence of early nodal and systemic spread. Systemic chemotherapy, adjuvant therapy, surgery and radiation have not improved disease prognosis or overall survival of IBC patients [3, 4].

## TUMOR MICROENVIRONMENT

The tumor microenvironment (TME) is the cellular environment in which the tumor exists and provides its niche for growth, progression, survival and evolution. The normal microenvironment provides crucial signaling to maintain appropriate tissue architecture, inhibit cell growth and suppress the malignant phenotype, and acts as a barrier to tumorigenesis [5]. Incorrect signals from TME can destabilize tissue homeostasis, initiate, promote, and push normal cells to malignant phenotype [5]. The TME is highly complex and dynamic, and includes blood vessels, immune cells, inflammatory cells, and components of the extracellular-matrix (ECM). TME is also rich in various cytokines, chemokines, ECM proteins, growth and angiogenic factors involved in autocrine but also paracrine cell signaling. The field of TME has expanded our understanding of cancer as more than a single factor driven disease. Rather, cancer biology involves complex reciprocal interaction and co-evolution between cancer cells and host stromal cells, interplay of soluble growth factors and chemokines as the key mediators involved in oncogenic signaling pathways of tumors. The list of anti-cancer therapies and clinical trials based on the role of the microenvironment in various types of cancers has grown long and it includes endostatin, Bevacizumab (Avastin), MK-2416, Anastroazole, Bay 43-9006, DX2400, Celecoxib, and PG545 etc. [5]. The majority of these are multikinase inhibitors blocking tumor cell growth pathways such as BRAF, Bcr-Abl, c-Kit, vascular endothelial growth factor receptor-1 (VEGFR-1), VEGFR-2, VEGFR-3, PDGFR and colony-stimulating factor-1 receptor [5]. Proteomic landscape of the breast cancer TME includes diverse factors involved in tumor growth, proliferation, metastasis, vascularity, evading cell death pathways and host immune system. In our previous study we demonstrated that inflammatory and invasive breast cancer TME is rich in OPG, chemokines such as urokinase-type plasminogen activator receptor (uPAR), Oncostatin M (OSM), IL-6 and GRO-α [6]. Here, we focus only on osteoprotegerin (OPG) as one of the factors present in the TME of inflammatory and invasive breast cancer cell lines, and discuss how it multitasks various functions to drive tumorigenesis.

## OSTEOPROTEGERIN (OPG)

**Figure 1:**
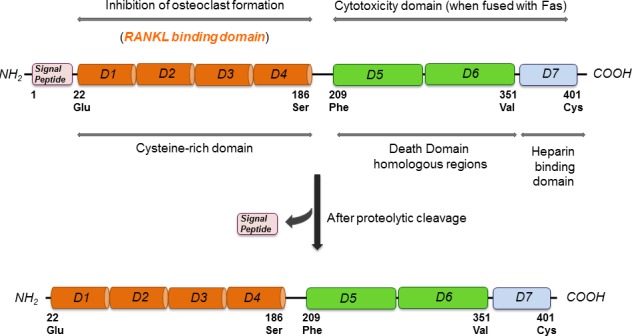
Molecular structure of OPG highlighting its different binding and functional domains

OPG was first identified by sequence homology to the tumor necrosis factor receptor (TNFR) family during a rat intestine cDNA sequencing project and was named based on its function (Latin: os bone, protegere to protect) [7]. OPG was independently discovered and alternatively named osteoclastogenesis inhibitory factor (OCIF) [8], TR1 [9], and follicular DC-derived receptor-1 (FDCR-1) [10], which are found to be identical to OPG. The human OPG gene is located at chromosome 8q23-24 and is composed of 401 amino acids [11]. The OPG protein comprises 401 amino acids of which 21 form a signal peptide [12] (Figure 1). Human and murine OPG consist of four cysteine rich pseudo repeats located in the N-terminal, two death domains, and a heparin-binding site located in the C-terminus and a 21 amino acid signal peptide [11]. Signal peptide is cleaved to generate a mature form of 380 amino acids (Figure 1). At the N terminus, there are four domains (D1-D4), which have cysteine-rich TNF receptor homologous motifs [12]. These motifs are required and are sufficient for binding to its major target, the receptor activator of nuclear factor (NFΚB) ligand (RANKL), and for inhibiting osteoclastic differentiation and activation [12]. At the C terminus, there are tandem death-domain homologous regions (D5 and D6) followed by a heparin-binding site (D7) [12] (Figure 1). More specifically at position 400, there is a cysteine required for homodimerization of the molecule (Figure 1). OPG is a secreted protein with no transmembrane or cytoplasmic domain, and is produced as a monomer (55-62 kDa) that undergoes homodimerization [11, 12]. OPG is secreted as a disulfide-linked homodimeric glycoprotein with four or five potential glycosylation sites, generating a mature form of OPG of 110-120 kDa [12]. The dimeric form of the protein exhibits a greater affinity for RANKL and a higher heparin-binding capacity than the monomeric form. The heparin binding site (D7) of OPG facilitates its binding to cell membrane-associated heparan sulfates. Heparan sulfates are expressed on the cell surface as heparan sulfate proteoglycans (HSPGs), and are involved in cell signaling, controlling cell behavior, actin cytoskeleton regulation, cell adhesion, and cell migration [13]. For biological use, D7, D5, and D6 are removed, and the remaining amino acids 22-194 OPG peptide is fused to the Fc domain of human IgG1 (OPG-Fc), which maintains the potent dimeric nature of full-length OPG and exhibits a significantly increased circulating half-life [12, 14]. OPG is highly expressed in the adult lung, heart, kidney, liver, thymus, lymph nodes and bone marrow [11]. OPG is syn­thesized by several cells including stromal cells, osteo­blasts, vascular smooth muscle cells, B lymphocytes, and articular chondrocytes [11].

## OPG AND BREAST CANCER

One of the first studies to characterize OPG revealed its expression in two human breast cancer cell lines, MDA-MB-436 and MCF-7 [15]. Further studies have confirmed the expression of OPG in breast cancer cell lines and tissues. The MCF-7, MDA-MB-231 and T47D human breast cancer cells lines were tested for OPG mRNA expression by real time PCR alongside 12 primary breast tumor samples [16]. OPG is expressed in 40% of breast cancers but not in normal breast tissue, and the TRAIL sensitive breast cancer cell line MDA-MB-436 produces sufficient levels of OPG to inhibit TRAIL-induced apoptosis *in vitro* [17]. OPG mRNA expression is upregulated in human breast cancer cell lines and tumor samples. The expression pattern of OPG was examined by immunohistochemistry in 400 invasive breast cancer tissue samples [18]. It was found that 40% of the invasive breast tumors expressed OPG with expression confined to tumor cells. It should be noted that the expression of OPG by tumor cells is not limited to the cancer cell expressing it but it has tremendous paracrine effect on the neighboring healthy cells.

Our study [6] for the first time, revealed that OPG is secreted and expressed at very high levels from the SUM1315MO2 invasive breast cancer cell line, as well as the SUM149PT and SUM190PT inflammatory breast cancer cell lines. OPG was secreted at a concentration of 500 pg/ml and 1100 pg/ml from SUM1315MO2 and SUM149PT, respectively [6]. Our study [6] demonstrated specific OPG staining in inflammatory breast cancer patient tumor sections apart from invasive breast cancer tumor sections which has been reported previously [18]. Heavy secretion of OPG led us to hypothesize that OPG directly or indirectly might be involved in inducing various oncogenic factors and thus contributing to the severity of the disease.

Breast cancer tissue is a heterogeneous system, mainly composed of tumor epithelial cells and stromal cells. The crosstalk between malignant and nonmalignant cells takes place in the breast TME at the primary site. This interaction, which can be *via* cytokines and chemokines, plays a major role in the various steps of breast cancer progression [19–23]. Therefore, the bidirectional crosstalk between the breast cancer cells and the tumor microenvironment components including immune cells, mesenchymal stem cells (MSCs), tumor associated fibroblasts (TAFs), fibroblasts, tumor vasculature, and extracellular matrix play vital roles in shaping tumor progression, aggressiveness and the bulk of the disease. This interaction, mainly driven by soluble secreted factors allows tumor cells to modify the stroma *via* tissue remodeling and gene expression and vice versa [22]. Therefore, defining the nature of the signals exchanged between the tumor microenvironment and the tumor cells should provide insights into how breast cancer develops and progresses, and may help to reveal therapeutic modalities based on intercepting the tumor-stroma crosstalk. Recruitment of the different components, including the MSCs and TAFs, is mediated by binding of different chemical messengers such as the TRAIL, TNF-α, TGF-β, chemokine (C-C motif) ligands, IL6, and platelet derived growth factor (PDGF) to their respective receptors. The MSCs are known to increase tumor progression, which is mediated by different cytokines such as OPG, TRAIL, IL-6, IL-8, CCL-5, CCL-2, and RANKL. These factors are known to increase the breast cancer development by regulating patient survival, bypassing apoptosis, invasion, migration and tumor angiogenesis [24]. OPG, TRAIL, and RANKL are significantly higher in tumor epithelial cells from patients with breast cancer than in epithelial cells of non-neoplastic breast tissues [25]. Moreover, the expression of OPG, TRAIL, RANKL, and RANK was significantly higher in spindle-shaped stromal cells from patients with breast cancer than in non-neoplastic tissue stromal cells [25].

## ROLE OF OPG IN METASTASIS

**Figure 2:**
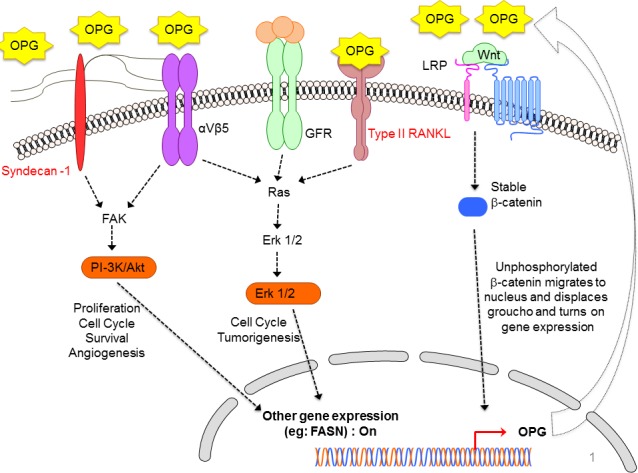
Schematic diagram depicting the diverse signaling pathways that are triggered or modulated by OPG OPG can bind to various cell surface receptors such as Syndecan-1, membranous RANKL, and αVβ5 integrin in order to trigger different cell survival pathways particularly, ERK, PI-3K/Akt pathway which results in expression of various cell survival favorable genes. Simultaneously, stable β-catenin of the canonical Wnt/β-catenin pathway, which is turned on in the majority of cancer cells, translocate to nucleus and turns on OPG gene expression. The OPG gets secreted out of the cell in the microenvironment, and can exert its paracrine and autocrine effects. The released OPG can affect the nearby healthy cells thus driving them towards tumorigenesis. The released OPG can also exert autocrine effects on the cancer cells by binding to cell surface receptors. The binding of OPG turns on the vicious cell survival signaling in cancer cells thus favoring their growth, proliferation, survival, angiogenesis [6].

Metastasis is an intricate multistep process, which is closely associated with worse prognosis of patients with tumors, and spread of the tumor. For example, bone metastasis is considered lethal and is an active area of research for potential therapeutic developments targeting bone cancer metastases. Bone metastases occur in more than 70% of breast cancer patients and cause severe skeletal complications such as fractures, spinal cord compression, bone pain, and hypercalcemia [26]. Patients with estrogen receptor-positive (ER+) breast cancer constitute a major clinical population who are at risk for bone metastases [26]. More than 50% of primary breast cancer cells express OPG and RANK, while RANKL could be detected only in 14-60% [25]. OPG is one of the important players of the OPG/ receptor activator of nuclear factor kappa-B (RANK)/RANK ligand (RANKL) triad (Figure 2), and is a secreted member of the TNFR superfamily of proteins [27]. OPG functions as an en­dogenous antagonist receptor that prevents the biological effects of RANKL, both membranous and soluble, and thus acts as an inhibitor of bone remodeling and resorption. Unlike RANK and RANKL, OPG does not have a transmembrane domain or cytoplasmic domain [28]. OPG is thus a decoy receptor, which interferes with the osteoclastic RANKL/RANK signaling to prevent bone loss [29].

The four cysteine rich pseudo repeats form an elongated structure and binds to one of the grooves of the active RANKL therefore preventing RANKL/RANK interaction and hence osteoclastogenesis. OPG production is modulated by sev­eral cytokines, vitamins, estrogens and other molecules, thereby modulating osteoclastogenesis and bone resorp­tion. OPG production is induced by 1α, 25-dihydroxyvi­tamin D3, estrogens, pro-inflammatory cytokines such as interleukin-1 (IL-1) and TNF-α as well as transforming growth factor-β (TGF-β), whereas parathyroid hormone (PTH) and glucocorticoids inhibit OPG production. OPG expression is modulated by biphosphonates in osteoblasts [30, 31].

Another study has also highlighted the possibility of bone derived OPG to increase survival of breast cancer cells that reach the bone microenvironment as part of the metastatic process thus promoting the breast cancer mediated bone osteolysis [32]. Aggressive breast cancer malignancies metastasize to bone and are associated with dysregulation of the RANK/RANKL/OPG pathway and can increase the RANKL/OPG ratio, which would favor excessive osteolysis [33]. OPG blocks the maturation of bone resorbing osteoclast cells in the bone microenvironment thus preventing bone resorption. OPG does not have a transmembrane domain or cytoplasmic domain [11]. A high affinity anti-RANKL monoclonal antibody denosumab (AMG162; bone antiresorptive drug) targeting the osteoblast/cancer cell interphase has been developed to prevent bone loss in prostate and breast cancer bone metastases by slowing down bone turnover, hence prostate and breast cancer growth on the skeleton [29]. Clinical phase III study showed that even patients with breast cancer without bone metastases but with reduced bone density due to treatment with aromatase inhibitors benefit from treatment with denosumab [34]. The treatment significantly increased bone density after 1-2 years compared with the placebo treated control group [34]. AMGN-0007 is a recombinant OPG construct and suppressed bone resorption when administered to multiple myeloma (MM) and breast cancer patients during a phase I clinical trial [35].

Knocking down OPG expression in triple-negative breast cancer cells led to a significant reduction in metastasis in the chick embryo metastasis model. A reduction in metastasis was observed from both a primary tumor and by intravenous injection of tumor cells, suggesting a direct impact of OPG on metastasis [2]. The discovery that OPG is a potent inhibitor of osteoclast activity and maturation initiated research into the possibility of using this molecule as a therapeutic agent for the treatment of a variety of conditions associated with increased bone resorption, including tumor-induced bone disease [36].

## OPG AS A SURVIVAL FACTOR

Discovery of OPG's ability to bind and inhibit the activity of TRAIL (TNF related apoptosis inducing ligand) opened an altogether new avenue for research suggesting that OPG production may provide cells with a survival advantage (Figure 2) [37]. TRAIL is produced in tumors by invading monocytes, inducing apoptosis in neoplastic cells sensitive to this cytokine. It has been shown in MDA-MB 436 and MDA-MB 231 cells that OPG produced by breast cancer cells enhances tumor cell survival by inhibiting TRAIL-induced apoptosis [18]. *In vitro* studies using a number of different tumor types have supported this hypothesis [18, 32, 38–40] but an undisputed functional link between OPG and cell survival in cancer has been not established to date. Resistance to apoptosis being a hallmark of cancer also correlates with aggressiveness of the tumor and poor prognosis. Wnt/ß-catenin, a major pathway of cell proliferation and growth has been shown to drive OPG expression thus leading to cell survival in colon cancer (Figure 2) [41]. In addition, the OPG produced by endothelial cells may increase survival through binding to TRAIL, thus helping the tumor cells to proliferate and increase metastatic bulk (Figure 2). One of the study highlighted the association of OPG expression in endothelial cells with increased tumor grade. The study also reported negative correlation between endothelial OPG and ER status of the tumors. OPG has been shown to be able to act as an autocrine survival factor for both breast and prostate tumor cells *in vitro* and this could also be a mechanism used by endothelial cells potentially favored by conditions within high grade tumors [42].

Apart from inducing angiogenesis and bypassing apoptosis by quenching TRAIL ligand, our study [6] for the first time demonstrated that OPG has the potential of reprogramming healthy human mammary epithelial spheres (HMEC), driving them towards tumorigenesis thus mimicking the scenario in breast cancer spheres (Figure 2). The addition of OPG to the normal HMEC growth media induced proliferation in the HMEC spheres. Another study [43] showed that intra-tibial tumors from the MCF-7 cells overexpressing OPG had an increase in cells staining positive for the proliferation marker Ki67. These observations together suggest that OPG can induce proliferation. Apart from a drastic increase in proliferation, few morphological changes were also induced in the control spheres. Aneuploidy, a hallmark of cancer, has been proposed to initiate tumorigenesis and is a remarkably common characteristic of tumor cells [44]. The increase in proliferation in HMEC spheres can be corroborated with the onset of aneuploidy markers such as IAK-1, Bub-1 and BubR1 [6].

Our goal was to decipher the cellular mechanisms of how OPG modulates and reprograms the normal mammary epithelial cells to a tumorigenic state thus suggesting promising avenues for treating IBC as well as highly invasive breast cancer with new therapeutic targets [6]. Our study also highlighted how OPG upregulates the phophorylated survival kinases such as Erk, Akt, GSK3β and p65 in HMEC spheres which contends the increased proliferation as seen in HMEC spheres in the presence of OPG (Figure 2) [6]. With DNA copy number variations (CNVs) in cancer cells having prognostic impact, it opens several avenues for therapeutic treatment and translational research. OPG selectively amplified the DNA copy numbers of AKT1, AURK1, EGFR, MYC and PAK1, CDK4 and downregulated tumor suppressive CDKN2A, PTEN and TOP2A genes (Table 1) [6]. OPG has been reported to exert its effects *via* OPG receptors, such as type II membrane forms of RANKL [45, 46], TRAIL [47] and heparan sulfate containing proteoglycans, such as syndecan-1 (Figure 2) [13, 48]. Interestingly, our study for the first time showed how one of the crucial breast cancer stem cell markers CD24 was upregulated in HMEC spheres in the presence of OPG which also supports the sustained proliferation in control spheres.

Recent studies have demonstrated OPG expression in breast cancer tissue samples and in a large cohort of invasive breast cancers (*n* = 400), 40% of samples showed OPG expression that was confined to tumor cells [18]. Interestingly, the gain in OPG gene copies was observed in 182 out of 934 tumors when the TCGA-2013 human breast invasive carcinoma data set was analyzed through the cBioPortal website [2]. The presence of an OPG copy number gain is a significant predictor of decreased overall survival or poorer prognosis in this cohort [2].

**Table 1:** Comparison of copy number variations in breast cancer cell lines, inflammatory breast cancer tissue from patient, and HMEC sphere cultures grown for long time in the presence of OPG rich microenvironment

Gene	SUM149PT	SUM1315M02	Patient sample	HMEC sp w/500pg/ml rhOPG
AKT1 (serine-threonine kinase)	✓	✓	✓	✓
AURKA (Aurora-A kinase)	✓	✓	✓	✓
CDK4 (cell cycle progression)	✓	✓	✓	✓
EGFR (surface receptor)	✓	✓	✓	✓
ERBB2 (RTK;oncogene)	✓	✓	✓	✓
MYC (regulator of transcripton)	✓	✓	✓	✓
PAK 1 (RhoGTPases)	✓	✓	✓	✓
PTEN (tumor sup.)	X	X	X	X
CDKN2A (CDK inhibitor)	X	X	X	X
RB1 (tumor sup.)	X	X	X	X
TOP2A	X	X	X	X

Red check marks denote the remarkably high gene expression of AKT1, AURKA, CDK4, EGFR1, ERBB2, PAK1 and MYC copy number in the DNA prepared from sphere cultures of SUM149PT, SUM1315MO2, inflammatory breast cancer tissue from patients, and HMEC spheres cultured for long time in the presence of human recombinant OPG (500pg/ml). Green crosses depict the downregulation of tumor suppressors (RB1 and PTEN), cycle regulator CDKN2A, and DNA repair enzyme topoisomerase DNA II alpha TOP2A in SUM149PT, SUM1315MO2, inflammatory breast cancer tissue from patients, and HMEC spheres cultured for long time in the presence of human recombinant OPG (500pg/ml).

## OPG AND ANGIOGENESIS

Angiogenesis is an essential step for breast cancer progression and dissemination. The development of new blood vessels in a tumor setting (angiogenesis) is conducted by numerous physiological and pathological stimuli. These stimuli can be various cytokines, chemokines, and growth factors. Molecular players of angiogenesis have been characterized since the early years of angiogenic studies, and one of the most prominent stimulating growing factors is certainly the vascular endothelial growth factor (VEGF) family. The most prominent member of this family, VEGF, is the foremost controller of physiological and pathological angiogenesis. An increasing number of reports now consider that OPG also has a function in other biological systems, including the vasculature [49–51]. It is highly possible that OPG produced either by the endothelial cells themselves or by the surrounding tumor cells, may result in increased endothelial cell survival and differentiation to form blood vessels, thereby facilitating tumor growth. OPG has been implicated in microvascular endothelial cell proliferation, migration, and induction of angiogenesis (Figure 2) [52].

Involvement of the heparin-binding domain D7 in OPG's proangiogenic activity has been suggested by McGonigle et al. 2009 [53]. OPG may interact with endothelial colony forming cells (ECFCs) through its binding to HSPG's, syndecan-1 (Figure 2), thereby exerting an anti-adhesive effect and promoting ECFC migration through a SDF-1/CXCR4 dependent pathway (as well as the MAPK and the Akt cascades), finally facilitating their recruitment to sites of neoangiogenesis [54, 55]. Our study [6] also corroborates the previous findings [42, 54, 56] highlighting OPG's important role in angiogenesis and neovasculogenesis in endothelial tube formation in an *in vitro* model of angiogenesis (Figure 2). When we used 500pg/ml and 1100pg/ml rhOPG, the length of the neovascular tubes were increased along with the number of branch points when compared to control media without rhOPG [6]. Quantitatively, conditioned media from SUM149PT and SUM1315MO2 adherent cell conditioned medium induced ~ 5-fold and 2.5 fold more branch points/field, respectively than HMEC medium [6]. However, OPG-depleted SUM149PT and SUM1315MO2 conditioned media reduced node formation by 36 and 51%, respectively [6].

## OPG AND ITS BINDING PARTNERS IN BREAST CANCER CELLS

**Figure 3:**
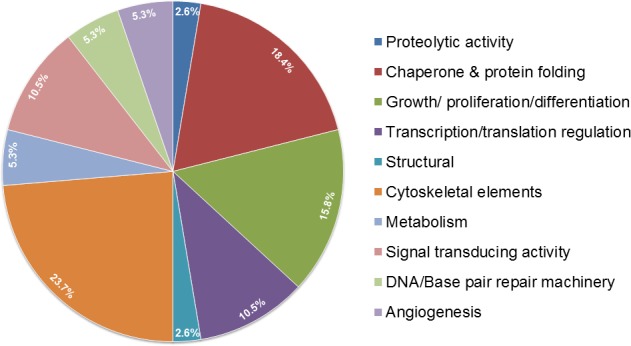
Pie chart depicting the binding partners of OPG from mass spectrometry analysis **A.** pull-down with anti-OPG antibody revealed a myriad of proteins involved in different cellular functions in inflammatory and aggressive breast cancer cells.

In our quest to understand more about the binding partners of OPG we performed mass spectrometry analysis. OPG pulled down proteins controlling cell proliferation (nucleolin, IQGAP1/3, proliferation inducing gene 32, proliferation inducing gene 44), proteins involved in transport and fusion (valosin and ATP binding cassette), and proteins involved in gene expression (Leucine rich PPR, DHX9 or DEAH, t-RNA synthetase) (Figure 3) (unpublished results). OPG pulled down many cytoskeleton elements such as myosin, filamin, keratin, ankyrin, vinculin, and α-actinin (Figure 3) (unpublished results).

Interestingly, immunoprecipitation of breast cancer cell extracts by OPG antibody revealed a major band at a molecular mass of 110 kDa (unpublished results). Mass spectrometry analysis revealed it to be the protein nucleolin (Figure 3) (unpublished results). Nucleolin is a multifunctional shuttling protein present in the nucleus, cytoplasm, and on the surface of some types of cells. Nucleolin is a major constituent of nucleoli in exponentially growing cells and functions in the organization of nucleolar chromatin, packaging of pre-rRNA, rDNA transcription, and ribosome assembly by shuttling between the nucleus and the cytoplasm [57–59]. Expression of nucleolin on the cell surface has been reported in HeLa cells [60], lymphoblastoid T cells [60], breast carcinoma cells [61, 62], lung [63], and laryngeal epithelial cells [64], and hepatocarcinoma cells [68]. Nucleolin is expressed on the surface of endothelial cells in angiogenic blood vessels [65]. The interaction between nucleolin and OPG in breast cancer cells adds another layer of complexity to how OPG could be manipulating functions at the nuclear levels, and these studies are ongoing in our lab.

The observation of cytoskeleton elements being pulled down validates with findings that have been reported previously [43]. Studies have investigated the mechanism whereby OPG could promote angiogenic behavior of endothelial cells. Human dermal microvascular endothelial cells (HuDMECs) treated with OPG were elongated with extensive actin networks compared to untreated cells [43]. This was due to cytoskeletal reorganization and cell spreading which was mediated by Focal Adhesion Kinase (FAK) as phosphorylation of FAK at tyrosine 397 was induced by treatment with OPG [43].

Valosin-containing proteins; also known as p97 or VCP, is an abundant ATPase which has the ability to use the energy derived from ATP hydrolysis to unfold client proteins [65]. VCP engages in a range of cellular processes such as ER-associated degradation (ERAD) that mediates the extraction of misfolded proteins across the ER membrane and their delivery to the proteasome. The ATPase valosin-containing protein (VCP; p97) is an essential regulator of protein degradation in multiple pathways and has emerged as a target for cancer therapy. VCP inhibition affects protein synthesis, eukaryotic initiation factor 2α (eIF2α) and mechanistic target of rapamycin complex 1 (mTORC1), and attenuates global protein synthesis. VCP inhibitors perturb intracellular amino acid levels, activated eukaryotic translation initiation factor 2α kinase 4 (EIF2AK4), and enhance cellular dependence on amino acid supplies, consistent with a failure of amino acid homeostasis [65]. Thus, depletion of VCP triggers cancer cell death in part through inadequate control of protein synthesis and amino acid metabolism, and allows it to have implications for the development of anti-cancer therapies [65, 66]. OPG's interaction with VCP in the context of invasive and inflammatory breast cancer opens up many interesting avenues to research the therapeutic implications.

OPG interestingly pulled down lipid metabolic enzyme, fatty acid synthase (FASN), which is a key enzyme of the fatty acid biosynthetic pathway in breast cancer cells (Figure 3) (unpublished results). FASN controls the process of producing de novo fatty acids from carbohydrate and amino acid-derived carbon sources [67]. FASN is a multifunctional polypeptide enzyme that produces saturated fatty acids, uses one acetyl-CoA and sequentially adds seven malonyl-CoA molecules to produce the 16-carbon saturated palmitic acid [67]. Overexpression of FASN has been strongly associated with many cancer types because it plays important metabolic roles in molecular pathways regulating cancer cell proliferation and tumor development [67]. Reduction in FASN enzyme activity by chemical inhibitors including orlistat, cerulenin and triclosan have been reported to remarkably decrease progression in various cancer cell types [68]. FASN is an attractive therapeutic target as it regulates neoplastic transformation, metastasis as well as angiogenic pathways manipulating tumor vascularity and cell proliferation [69]. It also serves as a potential diagnostic and prognostic biomarker as it is secreted in the blood of patients with breast, prostate, colon and ovarian cancers compared with normal healthy subjects [69]. In our recent study we demonstrated that compared to HMEC, SUM149PT and SUM1315MO2 breast cancer cells express increased level of FASN [70]. We have several interesting findings in the context of paracrine signaling in the OPG rich breast cancer microenvironment that drives carcinogenesis via inducing and sustaining inflammatory cycloxygenase-2 (COX-2) and lipogenic FASN in an invasive breast cancer setting (Figure 3) (unpublished results). Our lab findings reveal the synergistic anti-proliferative role of the COX-2 inhibitor celecoxib and FASN blocker C75 in aggressive metastatic breast cancer cells (Figure 3) (unpublished results).

## OPG IN CANCERS AND OTHER DISEASES

OPG has been reported to have connection with Prostate cancer, which is one of the malignancies that have great avidity to bone as advanced prostate cancer commonly metastasizes to bone leading to osteoblastic and osteolytic lesions [71]. Prostate cancer cells secrete OPG and *in vitro* OPG can protect the tumor cells from apoptosis *via* its ability to inhibit TRAIL and the apoptotic mechanisms it activates [18, 38, 40]. In contrast, *in vivo* OPG was shown to inhibit the survival of prostate cancer cells in bone [72, 73]. A potential role for serum OPG as a marker of early relapse in prostate cancer is suggested by the study of Eaton et al. 2004 [74]. Here, they compared serum OPG levels in 104 prostate cancer patients, ten cases of benign prostatic hyperplasia, and ten healthy young men [74]. The prostate cancer patients were divided into several groups: untreated patients with (i) organ confined or (ii) locally advanced disease, (iii) patients with advanced disease responding to androgen ablation and (iv) patients with early signs of disease progression. Serum OPG was found to increase in patients who progressed following androgen ablation, and this increase was detectable prior to elevation of the classical marker prostate specific antigen (PSA). The authors [74] suggest that serum OPG may not simply be a marker of advanced disease, but indicate changes in tumor cell survival and growth. The proinflammatory cytokine TNF-α has been demonstrated to drive OPG production in a variety of cell types and prostate cancers [75].

One of the major clinical features of multiple myeloma is the development of osteolytic bone disease and, over recent years, there is increasing evidence to suggest that the dysregulation of the RANK/RANKL/OPG system is important in the pathogenesis of myeloma bone disease. One study [76] has shown a subset of melanomas constitutively produce soluble OPG. OPG expression is regulated by the presence or absence of TNFR1 on melanomas, and melanomas drive OPG production in the absence of accessory cells through autocrine signaling *via* surface expression of mTNF. OPG levels are dramatically elevated in some colon carcinoma cells and that its secretion is potently upregulated by inflammatory cytokines such as TNF-α and IL-1 [77]. Increased serum OPG levels have been reported in patients with colorectal cancer (CRC)[41]. Interestingly, overexpression of OPG at the invasive tumor front plays a critical role in the initiation of progression and metastasis of CRC and high OPG level is a novel marker for recurrence after curative surgery for CRC [78]. The study also suggests OPG's potential to be used as a reliable biomarker to decide whether to use adjuvant chemotherapy in patients with CRC after surgery [78].

Ito et al. [79] examined the expression of OPG by gastric carcinoma cell lines and material from 103 cases of primary gastric carcinomas by gene expression analysis and immunohistochemistry, and related OPG expression to clinicopathological information such as tumor stage, depth of invasion, presence of lymph node metastasis and prognosis. They report a significant correlation between OPG expression and depth of tumor invasion, nodal metastasis and tumor stage, with strong OPG expression more frequent in stages III and IV than stages I and II. Mizutani et al. 2004 [80] demonstrated that serum OPG concentration is correlated with both tumor stage and tumor grade and that elevated serum OPG levels are predictive of early recurrence in patients with bladder carcinoma. These findings suggest that serum OPG concentration may have utility as a prognostic parameter in this setting.

OPG overexpression has also been related to poor prognosis for pancreatic cancer (PaC) and is a key modulator of metastasis and resistance to TRAIL-induced apoptosis [81]. Shi et al. 2014 showed increased OPG expression in PaC tissues compared with normal pancreas, and in PaC tumors [81]. OPG overexpression was associated with new-onset PaC-diabetes mellitus (PaC-DM) [81]. Patients with new-onset PaC-DM had a higher serum OPG level than those without diabetes mellitus [81]. These findings suggest that in at least a portion of PaC tissues, islet cells would face high OPG stress, and thus be more susceptible to damage than normal human islets. Taken together these studies also provide the rationale to consider OPG as a diabetogenic factor and most importantly as a candidate target for new approaches to retard the development of PaC-DM [81]. Toffoli et al. 2011 [82] found that OPG induces morphological alterations and reduction of islet function in mouse pancreatic islets. Serum OPG level may be used as a diagnostic tool and a prognostic variable for patients with muscle invasive bladder cancer. Future trials are required to elucidate its therapeutic role in such patients [83].

OPG is also involved in many other diseases unrelated to cancer. OPG was recently defined as an important cardiovascular (CV) marker in the general population with arterial stiffness, vascular calcification, and carotid intima media thickness [84]. OPG constitutes a novel biomarker with prognostic significance in patients with severe malaria. In addition, further studies are required to determine whether OPG plays a role in modulating malaria pathogenesis [85]. Circulating OPG levels are increased in patients with acute coronary syndrome [86] and enhanced expression has been found within symptomatic carotid plaques [87]. Elevated OPG levels have also been associated with the degree of coronary calcification in the general population as a marker of coronary atherosclerosis [88]. OPG levels were higher in obese than in normal subjects and HDL-C was also associated with OPG levels in obese women [89]. OPG has been reported to predict survival in patients with heart failure after acute myocardial infarction [90] to predict heart failure hospitalization and mortality in patients with acute coronary syndrome [86] and to be associated with long-term mortality in patients with ischemic stroke [91].

## OPG AND GENETIC POLYMORPHISM

The occurrence of single nucleotide polymorphisms (SNPs) in the OPG gene in association with breast cancer has been examined. Ney et al. 2013 [92] for the first time reported a significant association between the SNP rs3102735 (5′ near promoter region containing the minor allele C as well as for the homo- and heterozygous genotype with the minor allele C) of the OPG gene and the susceptibility of breast cancer in Caucasian populations. A 1.5-fold increased risk of breast cancer was associated with SNP rs3102735 [92]. Another study reported that the C allele of the OPG SNP rs2073618 and the T allele of the OPG SNP rs2073617 occurred more frequently in breast cancer patients [93]. The variant C allele of 950 T/C in the OPG promoter has been reported to play a major role as a genetic safe guard against progression in patients with prostate cancer [94]. The analysis of OPG gene variants C950T (promoter) and C1181G (exon 1) revealed that the presence of polymorphic 1181G/950T alleles and 950 TT/1181 GG genotypes may play a role in the development of bone disease [95]. Apart from breast cancer, genetic variations in form of polymorphisms in OPG and RANKL have also been associated with bone fractures in premenopausal patients with systemic lupus erythematosis (SLE) [96]. OPG/A163G polymorphism has been suggested to contribute to the genetic regulation of bone mineral density or bone turnover markers in Slovak population and thus could increase or decrease osteoporosis risk [97]. 163A/G (rs3102735) and 950T/C (rs2073617) polymorphisms of OPG have been evaluated in patients with pre-eclampsia (60 cases of early-onset severe pre-eclampsia and 91 cases of late-onset pre-eclampsia) [98].

Kwan et al., 2014 used a meta-analysis of GWAS from FIVE studies comprising > 10 000 individuals from European and Asian origin, and identified two genome-wide significant loci (8q23-q24.1 and 17q11.2) and one locus on chromosome 14 associated with OPG levels with near genome-wide significance [99]. In conclusion, they discovered that variants > 100 kb upstream of the gene encoding OPG are associated with variation in circulating OPG levels and identified another new significant locus on chromosome 17q11.2 as well as a suggestive locus on chromosome 14q21.2 associated with the trait [99]. They estimated that over half of the heritability of age-adjusted OPG levels could be explained by all SNPs studied [99].

## PERSPECTIVES

Despite the wealth of literature on OPG, there are many questions still unresolved, including the exact role of OPG in bone metastasis of breast cancer and the therapeutic potential of targeting OPG. Besides roles in cancer, OPG plays role(s) in bone metabolism, endometriosis, periodontal disease, thyroid disease and coronary heart disease [11]. Though OPG can modulate breast tumor growth and progression, future studies required to fully determine the mechanism of effect and overall outcomes of the different types of interactions required to determine whether strategies to block OPG signaling would be effective in blocking the development of primary breast tumors [43]. At this point OPG can no longer be considered solely in the bone microenvironment in breast cancer and caution must be exercised in the development of systemic treatment strategies aimed at increasing OPG levels. Localized delivery of OPG to the bone may be more appropriate to inhibit osteolysis linked metastatic breast cancer [43].

Conditionally replicating adenoviruses (CRAds) have been used as anticancer agents designed to infect and lyse tumor cells. In a murine model of osteolytic bone metastases of breast cancer, the CRAd armed with shortened OPG (sOPG)-Fc reduced tumor burden in the bone and inhibited osteoclast formation more effectively than an unarmed CRAd suggesting the role of OPG [17]. Breast cancer development in BRCA1/2 mutation carriers is a consequence of autonomous and nonautonomous cell factors, which serve as excellent targets for cancer prevention. BRCA-mutation carriers were reported to have lower mean values of free serum OPG in particular in BRCA1-mutation carriers compared with controls [100]. OPG may become a new biomarker and a target of treatment for patients with colorectal cancer since many studies have revealed the clinicopathologic significance of OPG expression by using clinical tissue samples from patients [78]. The effects of sustained expression of OPG using a recombinant adeno-associated viral (rAAV) vector in a mouse model of osteolytic breast cancer has clearly indicated the potential of rAAV-OPG therapy for reducing morbidity and mortality in breast cancer patients with osteolytic bone damage [101]. With all this development in the understanding of OPG, there might be more therapeutic avenues to manipulate OPG to predict and manage aggressive forms of breast cancer.

## Abbreviations

TME: tumor microenvironment
ECM: extracellular matrix
OPG: osteoprotegerin
TNFR: tumor necrosis factor receptor
TRAIL: TNF related apoptosis inducing ligand
IBC: Inflammatory breast cancer
RANKL: receptor activator of nuclear factor (NFΚB) ligand
OCIF: osteoclastogenesis inhibitory factor
FDCR-1: follicular DC-derived receptor-1
MSCs: mesenchymal stem cells
TAFs: tumor associated fibroblasts
PDGF: platelet derived growth factor
IL-1: interleukin-1
IL-6: interleukin-6
IL-8: interleukin-8
CCL-5: Chemokine (C-C motif) ligand 5
TNF-α: tumor necrosis factor- α
TGF-β: transforming growth factor-β
PTH: parathyroid hormone
PSA: prostate specific antigen
HSPGs: heparin sulfate proteoglycans
HuDMECs: Human dermal microvascular endothelial cells
FAK: Focal Adhesion Kinase
VEGF: vascular endothelial growth factor
PaC: pancreatic cancer
Pa-DM: PaC-diabetes mellitus
FASN: Fatty acid synthase
IQGAP1/3: IQ Motif Containing GTPase Activating Protein 1/3
CRAds: Conditionally replicating adenoviruses
CRC: colorectal cancer
ECFC: endothelial colony forming cells
AURK1/IAK1: Aurora A Kinase
EGFR: epidermal growth factor receptor MYC and PAK1
CDK4: Cyclin-Dependent Kinase 4
CDKN2A: Cyclin-Dependent Kinase Inhibitor 2A
PTEN: Phosphatase and tensin homolog
TOP2A: topoisomerase (DNA) II Alpha
